# Quantifying age and spatial variations of bone marrow elasticity with noncontact optical coherence elastography

**DOI:** 10.1117/1.JBO.30.12.124505

**Published:** 2025-09-10

**Authors:** Amandeep Singh, Manmohan Singh, Salavat R. Aglyamov, David Mayerich, Kirill V. Larin

**Affiliations:** a University of Houston, Department of Biomedical Engineering, Houston, Texas, United States; b University of Houston, Department of Electrical and Computer Engineering, Houston, Texas, United States

**Keywords:** optical coherence elastography, bone marrow, Rayleigh wave, femur, biomechanical properties

## Abstract

**Significance:**

The bone marrow is essential in immune regulation to maintain body homeostasis and to control the trafficking of stromal cells. A framework of connective tissue upholds bone marrow cells to maintain their mechanical and functional integrity. The biomechanical characterization of the bone marrow may provide useful insights for diagnosing hematologic diseases such as primary myelofibrosis. Optical coherence elastography (OCE) can measure the mechanical properties of tissues with high spatiotemporal resolution and may be well-suited for characterizing bone marrow elasticity.

**Aim:**

We demonstrate the quantification of the elastic modulus of bone marrow *ex vivo* at different locations along the diaphysis of mice femurs and compare the elastic modulus within different age groups of mice femurs.

**Approach:**

The femur bone marrow of CD1 mice, ∼12 weeks old (young adult), 24 weeks old (mature adult), and 1 year old (old adult), was imaged with OCE (N=4 femurs for each age group) to investigate the change in stiffness with age and location along the femur. A noncontact air-coupled ultrasound (ACUS) transducer induced elastic waves in the bone marrow, which were detected by phase-sensitive optical coherence tomography. The ACUS-OCE measurements were taken at three different locations along the diaphysis from the proximal end to the distal end to investigate the spatial stiffness variations.

**Results:**

The results show that the stiffness of femoral bone marrow increases significantly with age (p<0.001), but there was no significant difference in Young’s moduli among the locations for young ($\chi^{2}(2)=2.15$, p=0.33), mature ($\chi^{2}(2)=5.68$, p=0.058), and old ($\chi^{2}(2)=5.73$, p=0.056) mice femur samples.

**Conclusions:**

These findings show that OCE is promising for mapping the stiffness of the intact bone marrow and could be used for minimally invasive clinical applications.

## 1 Introduction

The bone marrow is a dense cellular tissue essential for producing and delivering blood cells into circulation, supporting tissue oxygenation, immunity, and coagulation.^1^ This soft tissue is located in the central cavity of long bones^2^ and is surrounded by a pore space of trabecular bones.^3^ This micro- and macro-structural environment is essential for the proper physiological function of pluripotent precursor cells^4^ and is responsible for the production of platelets, monocytes, and erythrocytes.^5^ The mechanical stiffness of marrow cells and their surroundings is a critical factor for the growth and proper differentiation of hematopoietic stem cell progenitors.^6^ Degenerative diseases such as diabetes,^7^ bone marrow aplasia,^8^ and osteoporosis^9^ are correlated with the accumulation of the adipose tissue within the bone marrow. In the case of myelofibrosis, excessive growth of the fibrillar extracellular matrix results in the development of the fibrotic bone marrow.^10^ These significant changes alter the biomechanical environment of the bone marrow and highlight the importance of exploring physiological dysfunctions in the bone marrow tissue with biomechanical analyses.^11^

A variety of methods, such as histological rheology, indentation, cavitation, and magnetic resonance imaging, have been utilized to characterize the biomechanical properties of the bone marrow to elucidate pathophysiological mechanisms. A mechanical rheometer was used to evaluate the viscosity of *ex vivo* porcine femur samples, and the effect of shear rate was investigated for human femur samples.^12^ A custom-built indentation instrument has been utilized to quantify the elastic modulus of the bone marrow in the femur of *ex vivo* pigs, and cavitation rheology has been demonstrated using a syringe pump integrated with a pressure sensor to quantify Young’s modulus.^13^ However, these contact-based methods increase the complexity of the experimental procedure and may introduce damage due to the delicate nature of bone marrow. Optical coherence elastography (OCE),^14^ which is the elastographic functional extension of optical coherence tomography,^15^ is a well-established technique for quantitative measurements of tissue biomechanical properties with several attractive features, such as label-free, in-depth, and high-resolution imaging.^16^^,^^17^ OCE generally entails inducing deformation in the tissue (contact or noncontact), imaging the response of tissue with OCT, and quantifying mechanical parameters with an appropriate mechanical model.^18^ In particular, wave-based OCE methods rely on mechanical wave speed measurements in tissues that can then be used for elastic quantification without any contact with the tissue, which preserves the integrity of delicate tissues such as the bone marrow and does not require any *a priori* information about the excitation force.^16^^,^^18^ Wave-based OCE methods have demonstrated numerous biomechanical evaluations in various fields, such as ophthalmology,^19^ dermatology,^20^ and developmental biology.^21^ Various noncontact excitation methods, such as a focused micro air-pulse,^22^ acoustic radiation force (ARF),^23^ and air-coupled ultrasound (ACUS),^24^^–^^26^ have been demonstrated in extensive applications for tissue mechanical characterization. In particular, ACUS combines the advantage of air-pulse and ARF for noncontact excitation with no coupling medium and tight spatio-temporal control for robust viscoelastic characterization with an appropriate mechanical model.^27^ OCE has the potential to evaluate the mechanical stiffness of the intact bone marrow to reveal new aspects of disease etiology in the bone marrow from a biomechanical perspective, which has not been thoroughly investigated.

In this work, we report the quantification of elasticity at different locations along the diaphysis of young, mature, and old normal mice femurs using noncontact ACUS-OCE. Validation and feasibility were tested on a 3D-printed mouse femur mold filled with agar. The microdeformations induced by ACUS resulted in Rayleigh wave propagation on the bone marrow tissue. The wave propagation along the bone marrow cavity was imaged using a phase-sensitive OCT system, and the Rayleigh wave speed was calculated by a distance-time slope method. Young’s modulus was estimated based on the Rayleigh wave speed.

## 2 Material and Methods

### 2.1 Sample Preparation

**Fig. 1:**
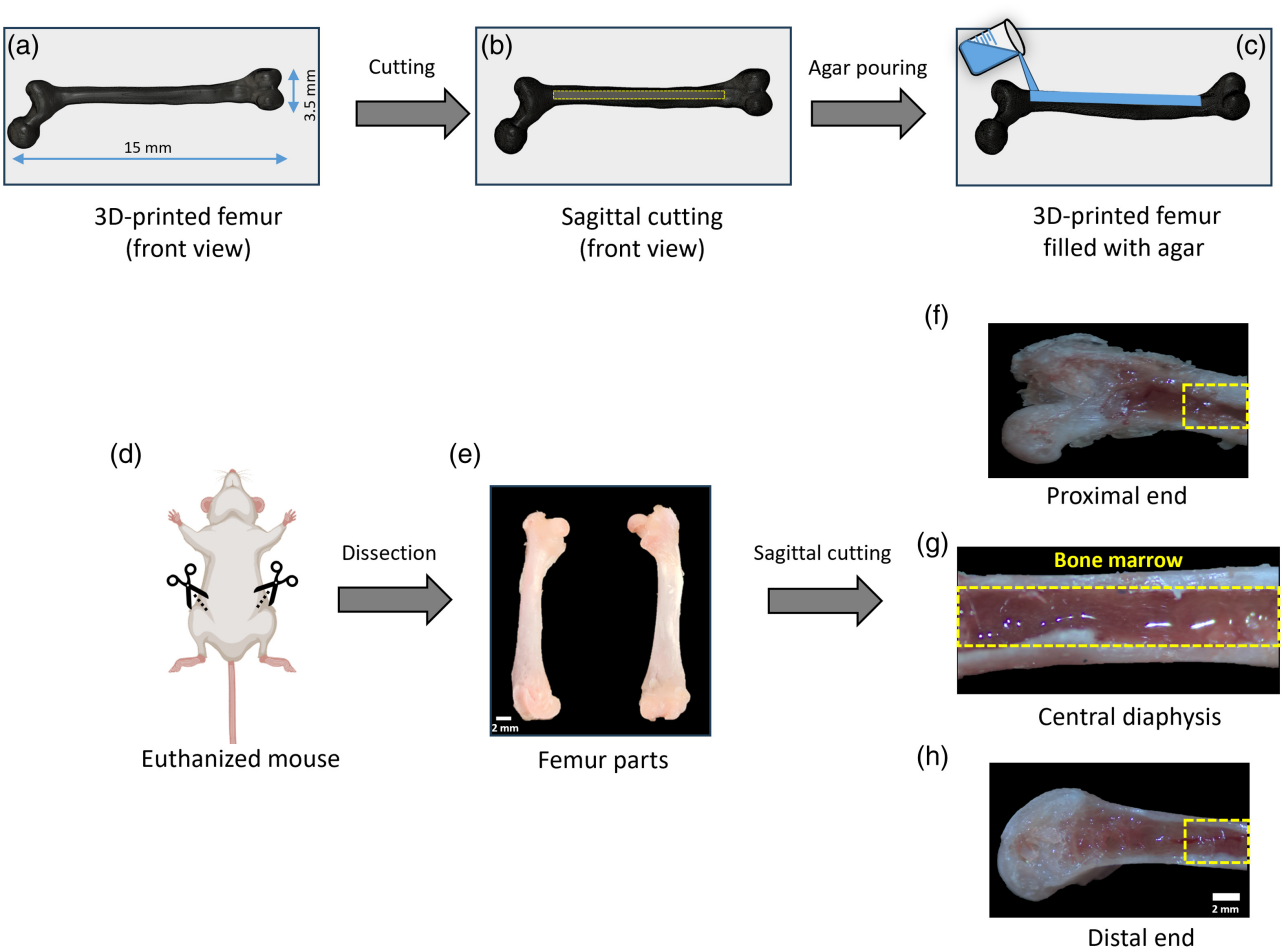
Front view of (a) the whole 3D-printed model of a mouse femur, (b) after a sagittal cut along the femur, and (c) the femur filled with agar. (d) Euthanized mouse for dissection. (e) An example of mice femurs after dissection. Microscopic image of (f) proximal end, (g) central diaphysis, and (h) distal end.

The proof-of-concept study was initially validated using a 3D-printed model of a mouse femur, as illustrated in Fig. 1(a). This model has a cavity along the central diaphysis, extending from the proximal end to the distal end, as shown in Fig. 1(b). Agar (1.5% w/v) solution was poured into the cavity along the femur, which is shown in Fig. 1(c). After solidifying, the elastic wave propagation was imaged at different locations along the 3D-printed mouse femur to cross-verify the feasibility of the current approach and evaluate the influence of the boundary conditions on wave propagation. The study was further extended to *ex vivo* mouse femurs of different age groups. The femur bone marrow of CD1 mice, ∼12 weeks old (young adult), 24 weeks old (mature adult), and 1 year old (old adult), was imaged with ACUS-OCE (N=4 femurs for each age group) to investigate the change in stiffness with age and location along the femur. The ACUS-OCE measurements were taken at three different locations along the diaphysis from the proximal end to the distal end to investigate the spatial stiffness variations. Before imaging, the mice were euthanized with ${\mathrm{CO}}_{2}$ inhalation followed by cervical dislocation, as shown in Fig. 1(d). The femurs were dissected out in a temperature-controlled dissection setup at 37°C, as illustrated in Fig. 1(e). The mechanical measurements were performed within 20 to 30 min post-dissection to minimize alterations in the native properties of the bone marrow tissue. In the current study, we have tried to maintain a consistent time window (sagittal cutting ∼3  min and ∼20  s for a single-elastography measurement at a single location). The sample handling protocol was designed to preserve native tissue morphology and hydration, which are primary factors influencing bulk mechanical behavior. The whole femurs were cut along the diaphyseal cross-section, and the samples were aligned in their respective positions for ACUS-OCE imaging, which is shown in Figs. 1(f)–1(h).

### 2.2 Wave Propagation in Biological Soft Tissue

**Fig. 2:**
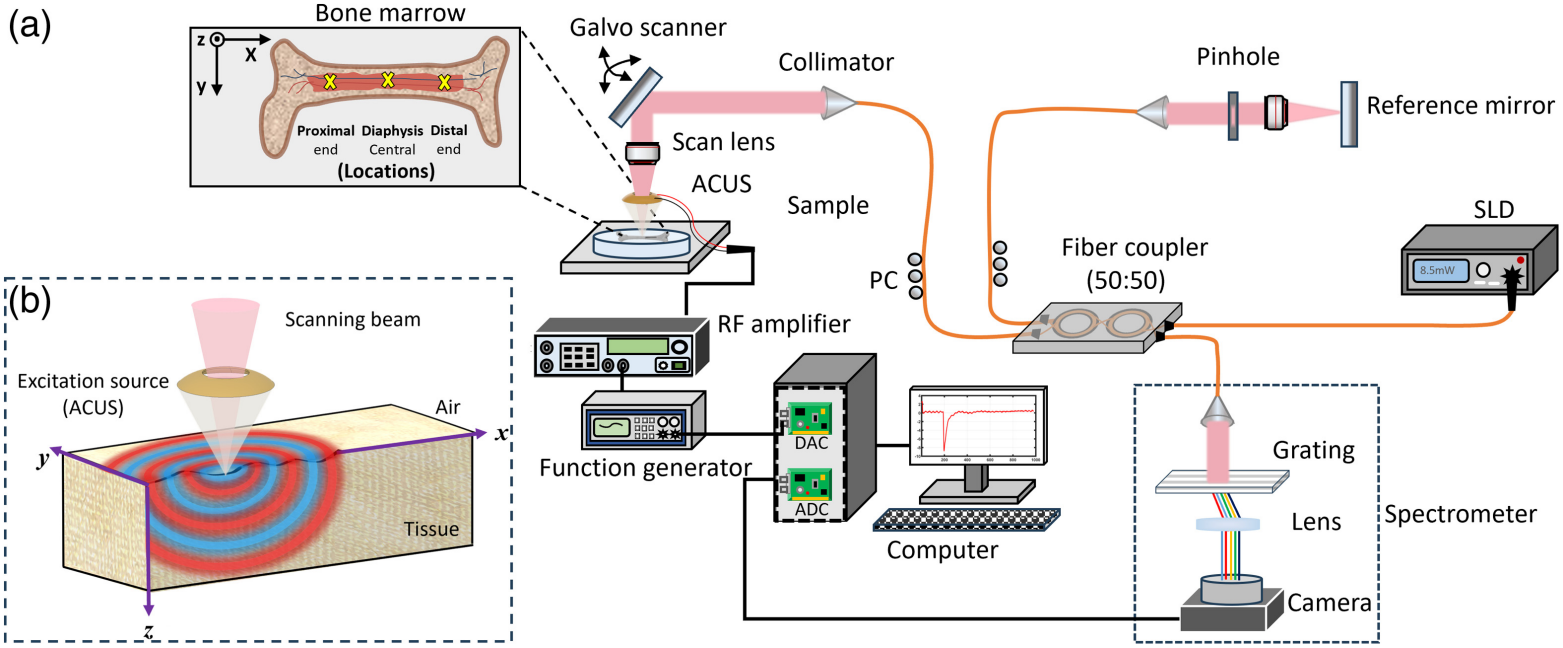
(a) Schematic of the ACUS-OCE setup for bone marrow elasticity imaging. ACUS: air-coupled ultrasound. ADC, analog-to-digital converter. DAC, digital-to-analog converter. PC, polarization control. RF amplifier, radio-frequency amplifier. SLD superluminescent diode. The inset shows the measurement locations along the mouse femur diaphysis. (b) Illustration of wave propagation on the tissue surface using ACUS excitation.

The elastic properties of soft biological tissue are commonly characterized by the shear modulus (μ) of the tissue.^18^ In wave-based OCE,^16^^–^^18^ a localized displacement is induced in the tissue, which then propagates as a mechanical wave, as illustrated in Fig. 2(b). The speed of surface (Rayleigh) mechanical waves $\left(C_{R}\right)$ in an incompressible medium can be directly linked to the shear modulus by $C_{R}=0.95\sqrt{\frac{\mu }{\rho }}$, where ρ is the mass density of the tissue. Biological tissue is often considered to be incompressible (Poisson’s ratio ∼0.5) because tissues are largely composed of water.^16^^,^^18^ Considering these assumptions, Young’s modulus (E) is related to the shear modulus by E=3μ.^16^^–^^18^ In this work, the Rayleigh wave model was used to quantify the elastic modulus due to the limited penetration depth of elastic waves. Before the experiment, different modulation frequencies were tested prior to selecting the 2 kHz excitation frequency. This frequency was chosen as it provided an optimal balance between wave penetration and effective excitation. Lower frequencies, with longer wavelengths, were more influenced by bone geometry and boundary effects, whereas higher frequencies were unable to induce measurable wave propagation. The measured wavelengths of elastic waves were less than the depth of the bone cavity, which supports our assumption of Rayleigh wave propagation with quasi-harmonic stimulation at a central frequency of 2 kHz for all samples.^28^^,^^29^

### 2.3 Experimental Setup

A schematic of the ACUS-OCE experimental setup and bone marrow samples is shown in Fig. 2(a). The system consists of a phase-sensitive OCT system based on the broadband superluminescent diode (S840-B-I-20; Superlum Diodes Ltd., Carrigtwohill, Ireland) operating at ∼840  nm center wavelength with a spectral bandwidth of ∼49  nm. The measured axial and lateral resolutions of the system were ∼9 and ∼8  μm in air, respectively. The displacement sensitivity was 0.28 nm. The system A-line rate was 25 kHz, corresponding to a temporal resolution of 40  μs. For the excitation, noncontact excitation was employed using a ∼1  MHz hemispherical ACUS transducer, which had a circular opening of ∼10-mm diameter with an outer spherical diameter of 20 mm.^25^ The focal length of the ACUS transducer was ∼20  mm, and it was aligned between the scan lens and sample so that the OCT and ACUS beams were co-focused. A 1-MHz sinusoidal signal was generated by a function generator (DG4162, RIGOL Tech, Beijing, China) and was modulated by five cycles of a 2-kHz square pulse with a 50% duty cycle. This signal was then amplified by a radio-frequency amplifier (A150, Electronics & Innovation, Rochester, NY) before driving the ACUS transducer.

### 2.4 OCE Measurements and Processing

The M-B mode^16^^,^^30^ imaging paradigm was used for acquiring the structural and motion data (2D along the x to z plane) in the bone marrow. The x-axis direction corresponded to the axis along the femur, i.e., horizontal in the bone marrow in Fig. 2(b), and the z-axis direction along the depth of the femur sample, as shown in Fig. 2(b). Each M-mode scan consisted of 1000 A-lines in time (40 ms) and 500 lateral points over ∼5  mm. A total of three locations (proximal end, central diaphysis, and distal end) were imaged for each sample. The data were processed in MATLAB^®^ R2023a (Mathworks, Inc., Natick, MA, United States). First, the surface was tracked, and the particle velocity, $v_{z}(t)$, was calculated by $v_{z}(t)=\frac{\lambda_{0}}{4\pi n\tau }\Delta \phi_{z}(t)$,^16^ where $\lambda_{0}=840\;\mathrm{nm}$ was the central wavelength of the light source, n was the refractive index of the tissue sample, τ=40  μs was the time interval, and $\Delta \phi_{z}(t)$ was the phase difference at each depth between consecutive A-lines. A finite impulse response filter in conjunction with a zero-phase filtering method was utilized to isolate the wave propagation at the excitation frequency (2 kHz). Motion artifacts due to the refractive index mismatch and surface motion were corrected.^31^ Then, the space-time map was reconstructed, and a linear regression method was used to calculate the slope of the elastic wave propagation in the space-time map and, hence, the elastic wave speed. Because the wave propagated in both directions away from the stimulation due to the confocal excitation setup, the speeds from both directions were averaged to improve accuracy.

### 2.5 Statistical Analysis

Statistical analysis was utilized to identify trends and make interpretations of OCE measurements as a function of femur location (proximal end, central diaphysis, and distal end) and age. We performed Kruskal-Wallis ANOVAs to analyze changes in bone marrow stiffness as a function of position for each age group and Mann-Whitney U tests to compare the differences in age groups at each position. Data are presented as box and whisker plots, where the boxes represent the interquartile range, the inscribed box is the mean, the horizontal line is the median, and the whiskers are the 25th and 75th percentiles. A two-sample t test was performed on the overall elasticity between the young, mature, and old mice.

## 3 Results

### 3.1 Wave Propagation in 3D-printed Femur Model

**Fig. 3:**
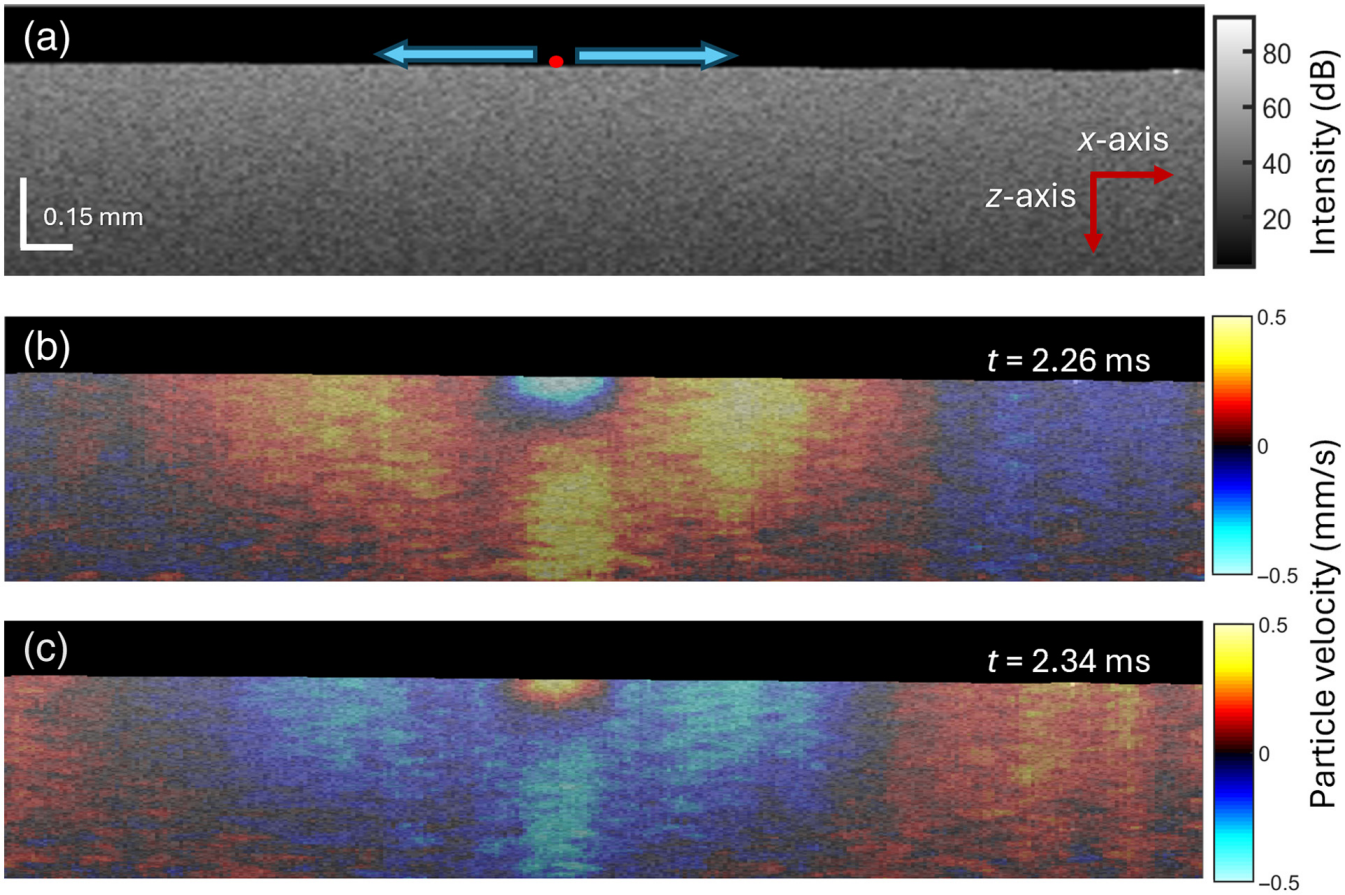
(a) An example B-mode OCT image of the 3D-printed femur filled with solidified agar (1.5%). Snapshots of the wave propagation at time (b) t=2.26  ms and (c) t=2.34 after the start of the quasi-harmonic excitation.

OCE measurements were performed in two configurations: one with the 3D-printed femur filled with solidified agar (1.5%) and another with the same agar concentration in a Petri dish (diameter: 35 mm, height: 11 mm) to assess the influence of boundary conditions and support our assumption of Rayleigh wave propagation. Figure 3(a) represents an example of a cross-sectional B-mode image of the 3D-printed femur filled with solidified agar (1.5%). The red dot represents the focal point of excitation of the ACUS transducer, and the blue bidirectional arrows represent the direction of wave propagation. The snapshots of wave propagation are illustrated in Figs. 3(b) and 3(c) at different time points after initiation of the excitation (t=2.26  ms and t=2.34  ms), respectively. The average speed of wave propagation along multiple locations (L=3, proximal, central, distal) of the 3D-printed femur was 3.52±0.10  m/s, which corresponds to Young’s modulus of 43.46±2.68  kPa with mass density $\rho =1060\;\mathrm{kg}/m^{3}$. The average speed of wave propagation in the culture dish was 3.44±0.09  m/s, which corresponds to Young’s modulus of 41.41±1.74  kPa. There was no significant difference (p=0.3 by Mann-Whitney U-test) in wave speed measurements between the two approaches. At the 2 kHz excitation, the wavelength was ∼1.75  mm, which is much shallower than the radius of the bone cavity (∼3.5  mm), thus reinforcing our assumption of Rayleigh wave propagation.^28^^,^^29^ To further validate the OCE results, standard uniaxial compression tests were performed on the same phantoms (Model 5943, Instron Corp., Norwood, MA, USA; 0.01 N pre-load, compression rate of 2  mm/min, end of test: 0.15 strain). The measured Young’s modulus was 41.51±3.26  kPa. The mechanical measurement derived from this study showed good agreement between OCE measurements (3D-printed femur, culture dish) and mechanical testing, reinforcing the reliability of the ACUS-OCE measurements. The measured speed was also in good agreement with previously published data^32^^–^^34^ for the same concentration of agar in tissue-mimicking phantoms with different techniques. The preliminary results demonstrated the successful implementation of the ACUS-OCE method to explore the biomechanical characteristics of the murine bone marrow.

### 3.2 Wave Speed Quantification at Different Spatial Locations Along the Mouse Femur

**Fig. 4:**
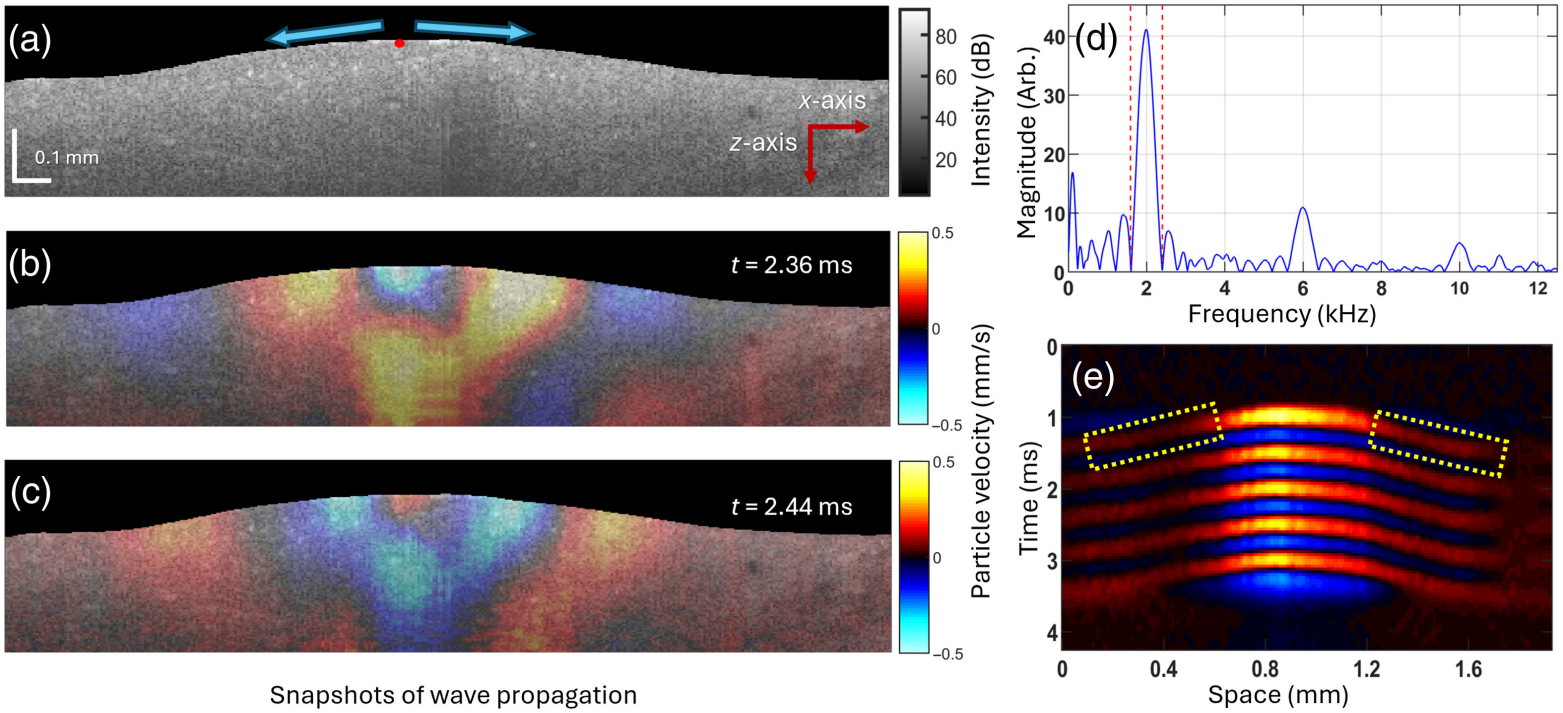
(a) An example B-mode OCT image of the bone marrow. Snapshots of the wave propagation at time (b) t=2.36  ms and (c) t=2.44 after excitation. (d) The spectrum of the axial particle velocity, where the red dashed lines are the lower and upper bounds used for filtering. (e) Space-time map of the wave propagation on the bone marrow surface.

Figure 4(a) represents an example of a cross-sectional B-mode image of the intact bone marrow in the x to z plane, where the red dot represents the point of excitation, and the blue arrows represent the direction of wave propagation. Figures 4(b) and 4(c) are snapshots of Rayleigh wave propagation at different time points after the excitation (t=2.36  ms and t=2.44  ms, respectively) to illustrate the wave propagation. Figure 4(d) shows the spectrum of the OCE-measured axial particle velocity (a temporal derivative of the displacement), and the red dashed lines are the upper and lower bounds used for filtering the axial particle velocity over time. The space-time map of the wave propagation is exemplified in Fig. 4(e), and the yellow dotted rectangle is the area where the wave speed was calculated. As described in Sec. 2, the wave speed was calculated in both directions of the excitation point, and the average was taken for each wave speed measurement for all samples.

For each age group, the wave speed was measured for N=4 femur samples at L=3 different locations (proximal end, central diaphysis, and distal end) along the femur from proximal to distal direction as summarized in Table 1.

**Table 1:** Wave speed measurements for different mice femurs at different locations.

Mice type (N=4)	Wave speeds at locations (m/s)		
	Proximal	Central	Distal
Young	1.53 ± 0.13	1.41 ± 0.11	1.52 ± 0.17
Mature	1.90 ± 0.05	1.58 ± 0.10	1.85 ± 0.16
Old	2.20 ± 0.24	1.90 ± 0.15	2.28 ± 0.19

**Fig. 5:**
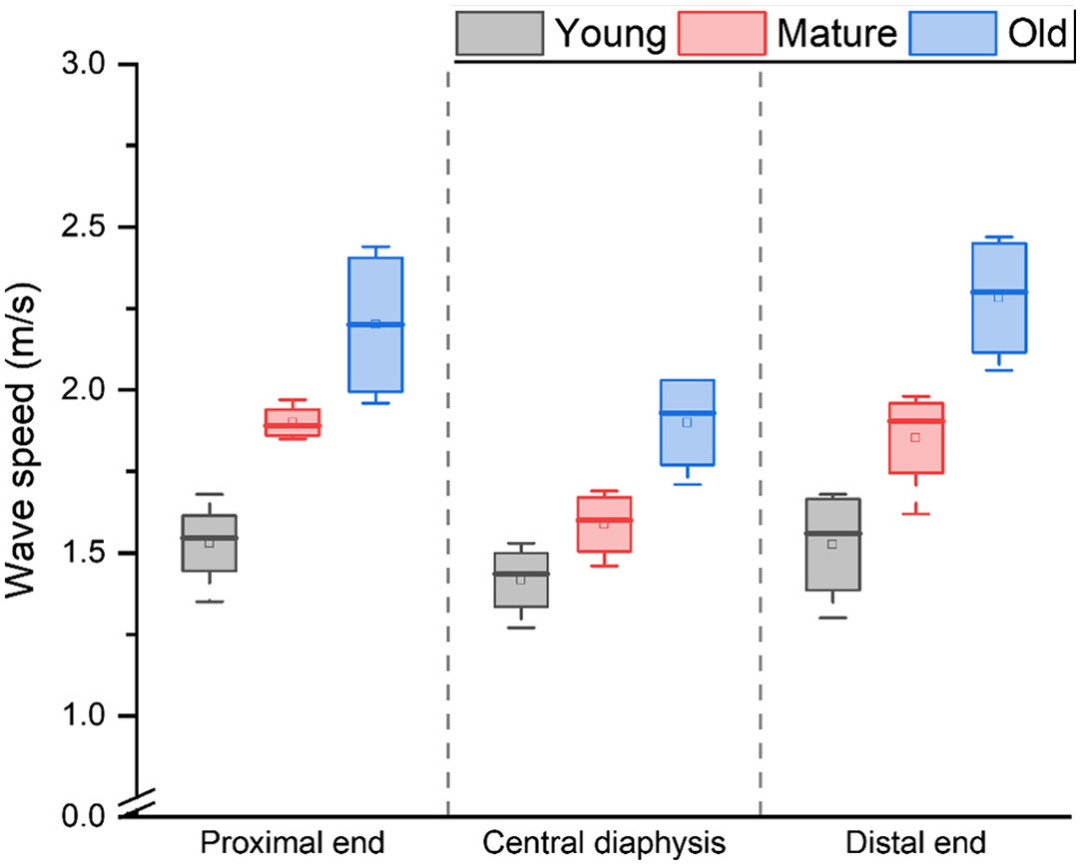
Comparison of wave speed values for young, mature, and old mice femur samples (N=4) at the proximal end, central diaphysis, and distal end locations. A p-value less than 0.05 was assumed to be statistically significant for all analyses.

Although the central diaphysis demonstrated some decrease in wave speed in comparison with other locations, there was no significant difference in wave speed among the locations for young ($\chi^{2}(2)=2.15$, p=0.33), mature ($\chi^{2}(2)=5.68$, p=0.058), and old ($\chi^{2}(2)=5.73$, p=0.056) mice femur samples. Figure 5 represents the quantifications of wave speeds for all the femur samples at different locations and for each age group.

### 3.3 Comparison of Young’s Modulus within Different Age Groups

**Fig. 6:**
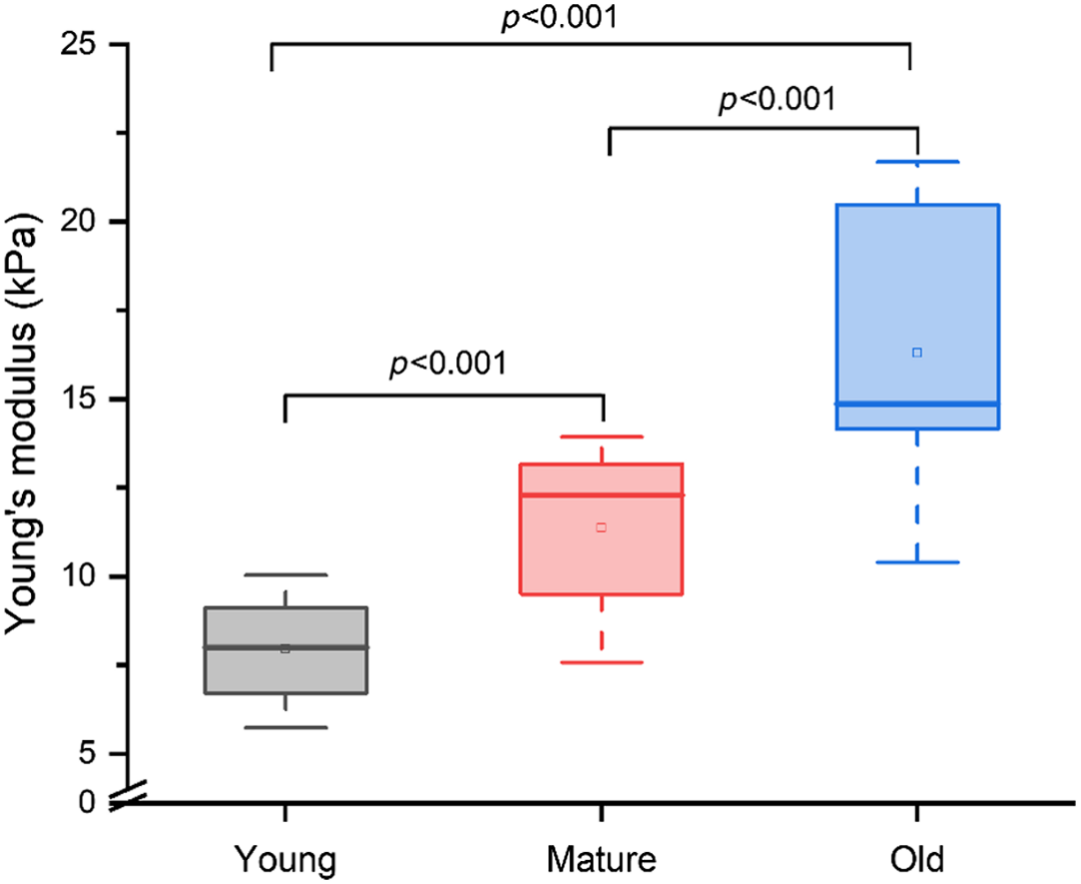
OCE-measured Young’s moduli for young, mature, and old mice femoral bone marrow.

The reported literature^35^^–^^37^ for the elastic modulus of bone marrow relies strongly on assumed Poisson’s ratios for calculation. These values typically vary from 0.2 to 0.5,^38^ and hence, the results vary up to 8%.^39^ In the current study, Poisson’s ratio and mass density were assumed to be 0.5^13^ and $1060\;\mathrm{kg}/m^{3}$^40^ for Young’s modulus estimation. Figure 6 represents the quantification of Young’s modulus for old, mature, and young mice femur samples. The average Young’s moduli estimated for the young, mature, and old mice were 7.12±1.33, 10.16±1.96, and 14.57±3.40  kPa, respectively, which were significantly different between all pairs (p<0.001). These findings highlight an age-dependent increase in bone marrow stiffness, consistent with physiological changes associated with aging.

## 4 Discussion

In the present work, we demonstrated the feasibility of ACUS-OCE for quantifying the stiffness of bone marrow at different locations along the femur of young, mature, and old mice. In addition, these results contribute to the literature on the change in the elasticity of the bone marrow during aging and are a critical first step toward utilizing OCE for nondestructive and rapid assessments of bone marrow viscoelasticity.

The previous methods for assessing the elastic properties of intact bone marrow have several limitations. These studies largely involve the extraction of the bone marrow to perform destructive measurements, which might not capture its *in situ* elasticity accurately. Rheological measurements^41^ depend upon the experimental parameters (plate temperature, frequency sweep, amplitude sweep, and contact area of probe), and any deviations in parameters vary the quantitative results. Mechanical indentation measurements^13^^,^^42^ involve critical parameters such as fixation, preload force, and probe geometry, which can all significantly affect the subsequent mechanical quantitation, resulting in poor reliability and accuracy. The present study overcomes these limitations with noncontact, fixation-free, label-free, and *in situ* measurements using ACUS-OCE.

The comparable wave speeds and corresponding Young’s moduli observed in both the 3D-printed femur and cultural dish phantom study configuration support the nonsignificant (p=0.3) influence of boundary conditions for femur geometry. This outcome validates our assumption of Rayleigh wave propagation, further supported by the fact that the excitation wavelength (∼1.75  mm) was considerably smaller than the characteristic cavity radius (∼3.5  mm), satisfying the shallow penetration criterion for Rayleigh waves.^28^ In addition, the choice of agar concentration (1.5% w/v) with higher stiffness gives a wavelength range (∼1.75  mm), which is greater than that of *ex vivo* bone marrow samples (∼1.23  mm). Consequently, in the case of stiffer bone marrow, the wavelength would remain within the surface-confined regime, preserving the validity of our Rayleigh wave assumption that reinforces the robustness of our OCE approach in assessing tissue stiffness, even in slightly stiffer biological environments such as native or pathological bone marrow.

In the current work, we have restricted the current studies by performing wave speed measurements along the femur diaphysis (x-axis direction) using a single excitation frequency. However, using broadband excitation and performing measurements in various meridional directions would reveal further information about viscoelastic and anisotropic properties. Because the femur bone is very stiff and the bone marrow is soft, the process of cutting the bone open along the femur diaphysis is critical to ensure the integrity of the bone marrow. It is important to note that the exposed surface of the bone marrow may exhibit some unevenness due to the technical challenges associated with bone cutting. This irregularity can introduce geometrical dispersion in surface wave propagation. To minimize its impact, measurements were performed in regions with the smoothest possible surface. The use of a decalcification method^43^ to soften the bone before cutting with a reagent such as formic acid, hydrochloric acid, or nitric acid would ease the measurements. However, the biomechanical properties of the bone marrow may also change, and investigating this change is an avenue of our future work. Nevertheless, the marrow was exposed in a controlled manner in this study, and future studies will be focused on developing more rigorous methods for exposing the bone marrow for *in situ* OCE bone marrow measurements.

Figure 6 shows that the elastic modulus of bone marrow increases significantly with age, which may be attributed to the change in marrow composition and adipose content with age.^6^^,^^12^ Literature reports^6^^,^^44^^,^^45^ suggest that the bone marrow undergoes age-related transformation from hematopoietic (red) to adipose-rich (yellow) tissue. Red and fatty marrow areas are not composed purely of either nonfatty cells or fat cells, respectively. Although fat content increases with age, other structural changes such as fibrosis, stromal remodeling, and greater integration with the surrounding bone may contribute to an overall increase in measured elasticity. This transformation is visually evident as a shift from red to yellow coloration and is accompanied by changes in tissue structure and mechanical properties.^6^ These findings suggest that marrow stiffness is not determined by fat content alone but reflects the combined effects of compositional and microarchitectural changes with age. The dehydration of bone marrow significantly influences the mechanical properties of the microenvironment of long bones. For example, dehydration increases the elastic modulus up to 22% (longitudinal) and 27.5% (transverse).^38^ Dehydration can be reduced by hydrating the marrow, e.g., with phosphate-buffered saline (PBS). However, PBS also decreases the elasticity of the bone marrow.^38^ Therefore, the elasticity imaging technique should be fast enough so that dehydration does not change the marrow environment and confound mechanical measurements. Thus, in the current study, we did not use PBS to prevent dehydration because ACUS-OCE performed a single elastography measurement in ∼20  s at a single location.

The measured elasticity of the bone marrow from the current approach cannot be directly compared with previous studies^13^^,^^46^ because experimental factors such as physiological temperature (rheology), loading (indentation), and contact area (AFM, rheology) need to be taken into consideration. The effective Young moduli for the bone marrow (porcine femur) lie in the range of 0.73 to 135.6 kPa from rheology measurements and 0.42 to 64.5 kPa using indentation methods.^13^ There are only a few limited studies describing the elastic properties of bone marrow, and in most cases, it is considered a viscous fluid. The viscoelastic quantification of bone marrow using ACUS-OCE with broadband excitation and a robust mechanical wave model, taking into account the bone marrow boundary conditions,^27^^,^^47^ is the next step of our work.

The current research is limited to bone marrow elastography; however, integrating the OCE set-up by different excitation sources (contact, noncontact) with appropriate excitation frequencies could be potentially effective for characterizing other regions such as the metaphysis and growth plate regions that play a significant role in maintaining the physicochemical environment around the marrow cells. It is important to mention that we used a constant assumed density for the bone marrow to compute Young’s modulus from the measured wave speed. This simplification may introduce inaccuracies, especially because the bone marrow density is known to vary with age, anatomical location, and fat content. Future work will aim to incorporate experimentally measured density values to improve the precision of elasticity quantification. Because the bone marrow is surrounded by rigid boundaries (compact bone), reverberant OCE^48^^,^^49^ (superposition of multiple shear waves) could be effective for characterizing bone marrow that is surrounded or intercalated with the bone, e.g., the trabecular region. We have recently demonstrated a noncontact form of reverberant OCE,^49^ and implementing this technique with ACUS excitation for high-resolution mechanical characterization of the bone marrow in 3D is the next step of our work.

## 5 Conclusion

We have demonstrated a noncontact, label-free, and nondestructive method for evaluating the elastic modulus of intact bone marrow using ACUS-OCE. This work had a two-fold aim: to quantify the change in stiffness in different age groups of the femoral marrow of mice and to assess the variation in elastic modulus in different locations along the femurs. The quantitative results from the current technique reflect the feasibility and potential of ACUS-OCE to detect spatial and age-related variations in the mechanical stiffness of the bone marrow. The present technique shows promise for future work to investigate the biomechanical properties of intact bone marrow under a variety of conditions and diseases, and our future work is focused on expanding this research with more robust mechanical imaging and quantitation of viscoelastic parameters.

## Data Availability

The data that support the findings of this article can be requested from the authors upon reasonable request.
